# Supplementary material for: Transcription near arrested DNA replication forks triggers ribosomal DNA copy number changes
Source: Nucleic Acids Res. 2025 Jan 29;53(3):gkaf014. doi: 10.1093/nar/gkaf014 (PMC11760980; doi:10.1093/nar/gkaf014)
Supplement: gkaf014_Supplemental_Files [file gkaf014_supplemental_files.zip › 1S-20241008-DSB repair in sir2 sae2.pdf]

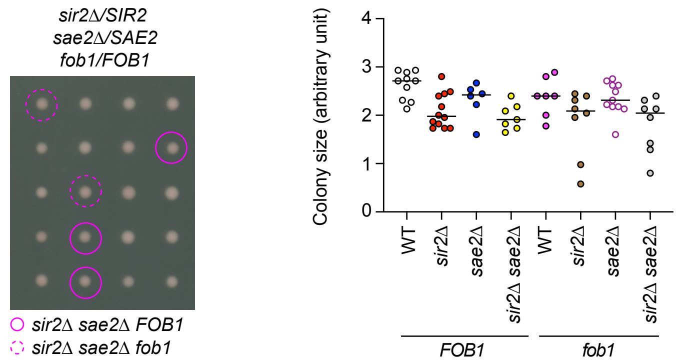

Supplementary Figure 1. Colony size analysis of *sir2*Δ *sae2*Δ strains.
Haploid clones were isolated by tetrad dissection of the diploid strain heterozygous for *sir2*Δ,*sae2*Δ , and *fob1*. Colonies of some genotypes were indicated. Colony sizes of different genotypes were analyzed. Bars represent median.
