# Supplementary material for: Transcription near arrested DNA replication forks triggers ribosomal DNA copy number changes
Source: Nucleic Acids Res. 2025 Jan 29;53(3):gkaf014. doi: 10.1093/nar/gkaf014 (PMC11760980; doi:10.1093/nar/gkaf014)
Supplement: gkaf014_Supplemental_Files [file gkaf014_supplemental_files.zip › 20241113-Supplementary Table 1.docx]

**Supplementary Table 1. *S. cerevisiae* strains used in this study**

| Name | Genotype |
| --- | --- |
| MSY360 | *MATa, NatNT2-GALL-FOB1, bar1::LEU2* |
| MSY375 | *MATa, NatNT2-GALL-FOB1, bar1::LEU2, ctf4∆::kanMX* |
| MSY476 | *MATa, NatNT2-GALL-FOB1, bar1::LEU2, MCD1-6His10FLAG::kanMX* |
| MSY769 | *MATa/α, fob1::LEU2/FOB1, rad52∆::hphMX/RAD52, sir2∆::kanMX/SIR2* |
| MSY937 | *MATa, NatNT2-GALL-FOB1, bar1::LEU2, sir2∆::hphMX, hml∆::kanMX* |
| MSY967 | *MATa, NatNT2-GALL-FOB1, bar1::LEU2, sir2∆::hphMX, hml∆::kanMX, ctf4∆::TRP1* |
| MSY988^a^ | *MATa, NatNT2-GALL-FOB1, bar1::LEU2, E-pro∆::GAL1/10p-URA3* |
| MSY1023 | *MATa, NatNT2-GALL-FOB1, bar1::LEU2, sir2∆::hphMX, hml∆::kanMX, rad52∆::klTRP1* |
| MSY1027 | *MATa, NatNT2-GALL-FOB1, bar1::LEU2, sir2∆::hphMX, hml∆::kanMX, sae2∆::klTRP1* |
| MSY1459 | *MATa, NatNT2-GALL-FOB1, bar1::LEU2, MCD1-6His10FLAG::kanMX, sir2∆::hphMX, hml∆::HIS3MX* |
| MSY1456 | *MATa, NatNT2-GALL-FOB1, bar1::LEU2, E-pro∆::GAL1/10p-URA3, MCD1-6His10FLAG::kanMX* |
| MSY1535 | *MATa, NatNT2-GALL-FOB1, bar1::LEU2, E-pro∆::GAL1/10p-URA3, rad50∆::kanMX* |
| MSY1541 | *MATa, NatNT2-GALL-FOB1, bar1::LEU2, E-pro∆::GAL1/10p-URA3, sae2∆::kanMX* |
| MSY1543 | *MATa, NatNT2-GALL-FOB1, bar1::LEU2, E-pro∆::GAL1/10p-URA3, rad52∆::hphMX* |
| MSY1583 | *MATa/α, fob1::LEU2/FOB1, sir2∆::hphMX/SIR2, mre11∆::klTRP1/MRE11* |
| MSY1585 | *MATa, NatNT2-GALL-FOB1, bar1::LEU2, sir2∆::hphMX, hml∆::kanMX, mre11∆::klTRP1* |
| MSY1594 | *MATa/α, fob1::LEU2/FOB1, sir2∆::hphMX/SIR2, sae2∆::klTRP1/SAE2* |
| MSY1872 | *MATa, NatNT2-GALL-FOB1, bar1::LEU2, sir2∆::kanMX, hml∆::hisMX, sgs1∆::hphMX, exo1∆::klTRP1* |

All strains are derivatives of W303, which is *ade2-1, ura3-1, his3-11, 15, trp1-1, leu2-3, 112, can1-100*, and *RAD5*.

^a^This strain was constructed from TAK2004a that carries ~80 rDNA copies and in which E-pro is replaced by the *GAL1/10* promoter.
